# Apatinib treatment efficiently delays biochemical-only recurrent ovarian cancer progression

**DOI:** 10.1186/s13048-021-00843-8

**Published:** 2021-07-12

**Authors:** Zhongyu Wang, Yake Huang, Ling Long, Li Zhou, Yan Huang, Lei Gan, Aimin Pu, Sufen Li, Rongkai Xie

**Affiliations:** 1 Institute of Cancer, Xinqiao Hospital, Third Military Medical University, Chongqing 400037, People’s Republic of China; 2 Department of Obstetrics and Gynecology, Xinqiao Hospital, Third Military Medical University, Chongqing 400037, People’s Republic of China; 3 Department of Obstetrics and Gynecology, Southwest Hospital, Third Military Medical University, Chongqing 400038, People’s Republic of China

**Keywords:** Ovarian cancer, Apatinib, Biochemical relapse, Anti-angiogenetic agents

## Abstract

**Background:**

Biochemical recurrence is defined as only rising CA-125 but no radiographic evidence of disease; noteworthily, it generally precedes the onset of clinical evidence. Now treatment strategies of biochemical recurrence ovarian cancer (OC) remain controversial. Apatinib as monotherapy or in combination with other chemotherapeutic agents has shown its effect in the treatment of some advanced malignancies. In our study, we focused on the efficacy of apatinib in recurrent OC, especially its clinical activity in biochemical-only recurrent OC patients.

**Methods:**

We retrospectively analyzed clinical material of 41 recurrent patients who had received apatinib monotherapy or apatinib plus chemotherapy between June 2016 and August 2018. Apatinib was administered at a 500mg daily dose. Response was determined according to measurable disease or serum carbohydrate antigen (CA)-125 levels. Progression-free survival (PFS) was estimated by Kaplan–Meier method.

**Results:**

All patients were evaluable, 19 (46.34%) had biochemical relapse and 22 (53.66%) had clinical relapse. The objective response rate (ORR) and disease control rate (DCR) in the overall population were 31.71% and 78.05%, respectively. The median PFS was 7 months (95% confidence interval 5.43–8.57). And in patients with biochemical-only relapse, the median PFS was 6 months, with ORR of 26.32% and DCR of 89.47%.

**Conclusions:**

Apatinib is a well-tolerated and effective agent to delay clinical progression of patients with biochemical-only recurrent OC. More important, our study shows the promising prospect for treating OC patients with asymptomatic biochemical relapse.

## Introduction

Ovarian cancer (OC) is the first-leading cause of death due to gynecological malignancies [1]. Due to a lack of specific symptoms, nearly 75% of patients are diagnosed with advanced OC at the initial visit, contributing to the low 5-year survival rate (approximately 20%) [2]. Cytoreductive surgery and platinum-paclitaxel combination chemotherapy are established as the primary treatments for advanced OC. However, the majority of patients who respond to initial treatment eventually experience a relapse and show low response to retreatment with cytotoxic therapy. Thus, the investigation of other effective treatment strategies remains a substantial clinical need.

Biochemical recurrence is defined as rising serum carbohydrate antigen (CA)-125 levels exceeding twice the upper limit of the normal range, without the disease being visualized on scans; noteworthily, it generally precedes the onset of clinical evidence by an average of 2 to 6 months [3, 4]. In such cases, the choice between either a watch-and-wait policy or early therapeutic intervention remains controversial. Thus, it urgently needs a breakthrough to optimize therapy, to delay clinical disease progression to the extent that would require intravenous chemotherapy.

Recently, the strategy of targeting angiogenesis has achieved success for the treatment of OC. Bevacizumab, a humanized monoclonal antibody that binds to all vascular endothelial growth factors (VEGF), has been approved by the European drug administration for the treatment of advanced ovarian carcinoma, specifically for recurrent platinum-sensitive or -resistant OC [5].

Apatinib is an oral vascular endothelial growth factor receptor 2 (VEGFR-2) inhibitor that inhibits tumor angiogenesis by blocking downstream signaling [6]. In China, apatinib has shown its effects in the third-line treatment of advanced gastric adenocarcinoma and adenocarcinoma in the gastric-esophageal junction, and apatinib monotherapy has been approved as a third-line treatment for patients with metastatic gastric cancer and as a second-line treatment for patients with advanced hepatocellular carcinoma by the Food and Drug Administration of the Peoples Republic of China (CFDA) [7–9]. Some studies also suggested the use of apatinib in other advanced malignancies, including breast cancer, liver cancer, small cell lung cancer, non-small cell lung cancer, colorectal cancer, sarcoma and osteosarcoma [10–17]. In additional, apatinib combined with anti-PD-1 inhibitor had promising antitumor activity in patients with advanced cervical cancer and extensive small cell lung cancer [18, 19]. Therefore, in OC, apatinib has got increasing attention about its efficacy and safety [20–24]; however, to our knowledge, apatinib’s efficacy in recurrent cancer, especially in biochemical-only recurrent OC, is still unknown. Hence, we conducted this study to report the efficacy of apatinib in recurrent OC, and aimed to preliminarily assessing the outcome of apatinib monotherapy in biochemical recurrent OC patients.

## Materials and methods

### Patients

In this retrospective study, we gathered the material of patients diagnosed with OC via pathologic evaluation, who had received apatinib monotherapy or treatment with apatinib plus chemotherapy between June 2016 and August 2018 in the first and second affiliated hospital of the Third Military Medical University. Patients were considered eligible for analysis if: 1) they received at least one line standard chemotherapy after debulking surgery and 2) relapse of disease was demonstrated by a measurable tumor or by an elevated level of CA-125. The study also enrolled patients who were intolerant to chemotherapy. Additional inclusion criteria included appropriate renal, hepatic, and hematopoietic function and an Eastern Cooperative Oncology Group performance status (ECOG PS) of 0–2. Patients with a history of bleeding, hypertension, ischemic cardiovascular disease, or proteinuria were ineligible for this study. The patients participating in this study provided written informed consent before study initiation.

### Treatments

The administration of apatinib as monotherapy or in combination with chemotherapy was determined according to different patient needs. Apatinib monotherapy was applied to patients who were no longer tolerant to chemotherapy or patients with biochemical recurrence of disease. Patients with relapse of a measurable tumor received treatment with apatinib and chemotherapy based on a taxane or etoposide. The recommended initial dose of apatinib was 500mg, po qd, half an hour after a meal at the same time every day. Chemotherapy was given simultaneously with apatinib for 28 days of one cycle. If intolerable toxicity occurred, the patient was informed to gradually reduce the dose to 250mg or discontinue the medication.

### Evaluation

The first evaluation for clinical efficacy and safety was performed at the end of the first cycle. Subsequently, the interval of assessment changed to every two cycles. Treatment efficacy in patients with measurable disease was assessed by CT, MRI, and ultrasound scans. Complete remission (CR), partial remission (PR), stable disease (SD), and progressive disease (PD) were evaluated according to the Response Evaluation Criteria in Solid Tumors (RECIST) (Version 1.1). Responses of biochemical recurrent patients were determined through serum CA-125 levels. The CA-125 definition for response rate was based on the Rustin criteria [25]. A reduction of CA-125 to normalization that was maintained for at least 4 weeks was defined as CR, a 50% reduction as PR, a 25% increase as PD, and a situation beyond the above criteria was recognized as SD [26]. CR plus PR was categorized as objective response rate (ORR) and CR, PR, plus SD was defined as disease control rate (DCR). The period from initial treatment to disease progression or death was defined as progression-free survival (PFS). Drug-related adverse effects were evaluated and graded according to the National Cancer Institute Common Terminology Criteria for Adverse Events (NCI-CTCAE) (version 4.0).

### Statistical analysis

The percentage method was used for categorical variables and drug safety analysis. PFS was analyzed by the Kaplan–Meier method, and the corresponding figures were drawn using GraphPad Prism 8.0 software (GraphPad Software Inc., San Diego, CA, USA). A P value < 0.05 was regarded statistically significant.

## Results

### Patient characteristics

Between June 2016 and August 2018, a total of 41 advanced OC patients were enrolled in this study. The median age was 53 years (range, 36–67). Most patients (33, 80.48%) presented with stage IIIC (Federation International of Gynecology and Obstetrics FIGO) disease. The histological type included serous carcinoma (high grade 70.73%; low grade 21.95%), mucinous carcinoma (4.88%), and endometrioid carcinoma (2.44%). Of 41 patients, 14 (34.15%) had optimal debulking surgery, whereas the remaining 27 (65.85%) received suboptimal debulking surgery. All were tumor recurrent patients after previous therapy; of these, 19 (46.34%) patients had biochemical recurrence and 22 (53.66%) patients had a visible tumor. Among the included patients, 29 (70.73%) had received 1–2 lines of chemotherapy and 12 (29.27%) had received 3–5 lines of treatment before participating. In this study, 13 of 41 (31.71%) patients were treated with apatinib in combination with chemotherapy and 28 patients (68.69%) received apatinib monotherapy, and the 28 patients included not only 19 patients with biochemical recurrence but also 9 patients with clinical recurrence who were no longer tolerant to chemotherapy. Moreover, most patients (75.61%) had good Eastern Cooperative Oncology Group performance status (ECOG PS) (0), and patients with ECOG PS of 1 and 2 accounted for 14.63% and 9.76%, respectively. Detailed baseline clinical characteristics of the patients are shown in Table 1.

**Table 1:** Patients’ baseline clinical characteristics

Characteristics	n (%)
**Median age (rang)(years)**	53 (36–67)
**FIGO stage**	
IIIA	1 (2.44%)
IIIB	3 (7.32%)
IIIC	33 (80.48%)
IV	4 (9.76%)
**ECOG PS**	
0	31 (75.61%)
1	6 (14.63%)
2	4 (9.76%)
**Histology type**	
High-grade serous carcinoma	29 (70.73%)
Low-grade serous carcinoma	9 (21.95%)
Mucinous carcinoma	2 (4.88%)
Endometrioid carcinoma	1 (2.44%)
**Debulking surgery**	
Optimal	14 (34.15%)
Suboptimal	27 (65.85%)
**Biochemical recurrence**	
Yes	19 (46.34%)
No	22 (53.66%)
**Previous chemotherapy lines**	
1–2	29 (70.73%)
3–5	12 (29.27%)
**Treatment regimen**	
Combined chemotherapy	13 (31.71%)
Monotherapy	28 (68.29%)

FIGO, International Federation of Gynecology and Obstetrics; ECOG PS, Eastern Cooperative Group Performance Status

### Efficacy

**Fig. 1:**
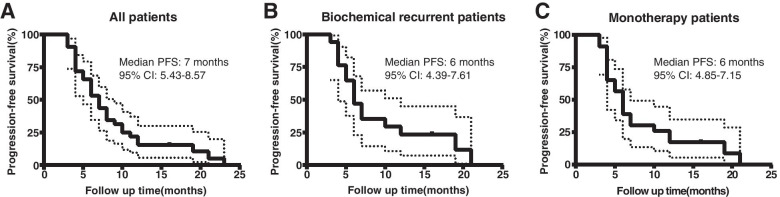
Kaplan-Meyer survival curve for estimating progression-free survival (PFS) in overall population (**A**), patients with biochemical relapse (**B**) and patients with apatinib monotherapy (**C**)

**Fig. 2:**
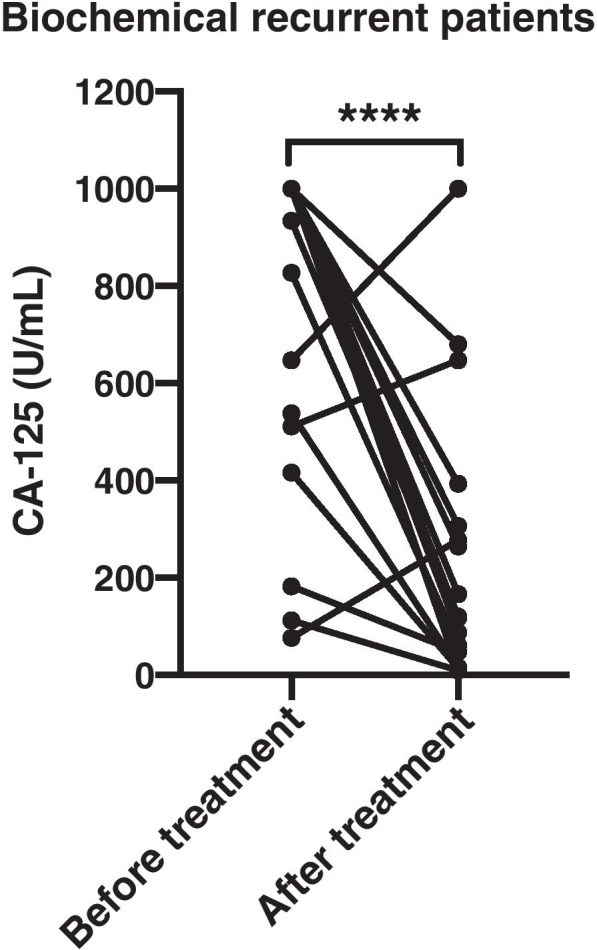
The data of CA-125 in patients with biochemical replase

During a follow up in July 2019, all patients were evaluated. Efficacy analysis indicated that none of the 41 patients achieved Complete remission (CR), 13 patients achieved partial remission (PR), 19 patients maintain stable disease (SD), and 9 patients had progressive disease (PD), resulting in an objective response rate (ORR) of 31.71% and a disease control rate (DCR) of 78.05% (Table 2). The median progression-free survival (PFS) was 7 months (95% CI 5.43–8.57, Fig. 1A). Among the patients with biochemical relapse, ORR and DCR were 26.32% and 89.47% respectively (Table 2), with median PFS of 6 months (95% CI 4.39–7.61, Fig. 1B). Based on apatinib-monotherapy patients, ORR and DCR were 21.43% and 82.14% respectively (Table 3), with median PFS of 6 months (95% CI 4.85–7.15, Fig. 1C). In addition, we also analyzed the CA-125 levels of biochemical recurrent patients including before and after treatment. The results displayed that the levels of CA-125 in those patients decreased significantly after apatinib monotherapy (Fig. 2).

**Table 2:** Treatment response in biochemical or imageological patients

	**Biochemical response (n, %)**	**Imageological response (n, %)**	**Overall response (n, %)**
Complete response (CR)	0 (0%)	0 (0%)	0 (0%)
Partial response (PR)	5 (26.32%)	8 (36.36%)	13 (31.71%)
Stable disease (SD)	12 (63.16%)	7 (31.82%)	19 (46.34%)
Progressive disease (PD)	2 (10.53%)	7 (31.82%)	9 (21.95%)
Objective response rate (ORR)	5 (26.32%)	8 (36.36%)	13 (31.71%)
Disease control rate (DCR)	17 (89.47%)	15 (68.18%)	32 (78.05%)

**Table 3:** Treatment response to apatinib monotherapy or combination therapy

	**Monotherapy response (n, %)**	**Combination therapy response (n, %)**	**Overall response (n, %)**
Complete response (CR)	0 (0%)	0 (0%)	0 (0%)
Partial response (PR)	6 (21.43%)	7 (53.85%)	13 (31.71%)
Stable disease (SD)	17 (60.71%)	2 (15.38%)	19 (46.34%)
Progressive disease (PD)	5 (17.86%)	4 (30.77%)	9 (21.95%)
Objective response rate (ORR)	6 (21.43%)	7 (53.85%)	13 (31.71%)
Disease control rate (DCR)	23 (82.14%)	9 (69.23%)	32 (78.05%)

### Safety

Adverse reactions were assessed and summarized in Table 4. In general, most patients were tolerant to apatinib without any grade 4 adverse events (AEs). The most common grade 1–3 AE was hand-foot syndrome (46.4%). Other common AEs were mucositis (41.5%), anorexia (39.0%), fatigue (39.0%), proteinuria (36.6%), hypertension (34.1%), and thrombocytopenia (26.8%).

**Table 4:** Adverse events

Adverse events	Grades				
	**1** **(n, %)**	**2** **(n, %)**	**3** **(n, %)**	**4** **(n, %)**	**Total** **(n, %)**
**Hand-foot syndrome**	5 (12.2%)	9 (22.0%)	5 (12.2%)	0 (0%)	19 (46.4%)
**Mucositis**	3 (7.3%)	6 (14.6%)	8 (19.5%)	0 (0%)	17 (41.5%)
**Anorexia**	12 (29.3%)	3 (7.3%)	1 (2.4%)	0 (0%)	16 (39.0%)
**Fatigue**	4 (9.8%)	7 (17.1%)	5 (12.2%)	0 (0%)	16 (39.0%)
**Proteinuria**	11 (26.8%)	4 (9.8%)	0 (0%)	0 (0%)	15 (36.6%)
**Hypertension**	5 (12.2%)	6 (14.6%)	3 (7.3%)	0 (0%)	14 (34.1%)
**Pain**	6 (14.6%)	4 (9.8%)	1 (2.4%)	0 (0%)	11 (26.8%)
**Thrombocytopenia**	6 (14.6%)	3 (7.3%)	2 (4.9%)	0 (0%)	11 (26.8%)
**Neutropenia**	2 (4.9%)	2 (4.9%)	1 (2.4%)	0 (0%)	5 (12.2%)
**Transaminase increased**	1 (2.4%)	3 (7.3%)	1 (2.4%)	0 (0%)	5 (12.2%)
**Diarrhea**	1 (2.4%)	2 (4.9%)	0 (0%)	0 (0%)	3 (7.3%)

## Discussion

Traditional therapies for OC, including debulking surgery and chemotherapy, cannot yield a good response rate in all relapsed OC patients. Efforts to understand OC biology have facilitated the development of new targeted antineoplastic agents. In cancer, angiogenesis contributes to tumor growth and invasion [27]. Multiple growth factors play proangiogenic roles, including VEGF, epidermal growth factor (EGF), and platelet-derived growth factors (PDGF); of these, the VEGF pathway is pivotal in angiogenesis [28]. Bevacizumab, a monoclonal antibody targeting VEGF-A, has been approved for the treatment of recurrent platinum-sensitive or -resistant OC [5]. Several multitargeted receptor tyrosine kinase inhibitors (TKIs), such as imatinib, cediranib, sorafenib, sunitinib, and pazopanib, target VEGFR, PDGFR, and FGFR. Many of these inhibitors have been or are being evaluated in clinical trials in OC, and some agents have exhibited inhibitory effects [5].

Most of the studies demonstrating clinical activity in advanced OC are small case reports. Only two prospective studies tested the efficacy of apatinib treatment in advanced OC. One is a single arm clinical study, which assessed the efficacy and safety of apatinib as monotherapy in patients with recurrent platinum-resistant epithelial OC [21]. The ORR and DCR in 28 patients receiving apatinib 500mg daily were 41.4% and 68.9%, respectively, and the median PFS and OS were 5.1 months and 14.5 months [21]. In this study, in the 28 monotherapy patients, the ORR was 21.43% and DCR was 82.14%, the median PFS was 6 months. The other study assessed the activity of apatinib plus etoposide in the treatment of patients with platinum-resistant or -refractory OC, showing an ORR of 61% and DCR of 97% of 31 patients in the per-protocol population [20]. The ORR in our combination therapy population with 13 patients was 53.85%, and the DCR was 69.23%. And the effectiveness of the above two prospective studies were more significant than this study. This was probably due to the small sample size, as well as to the retrospective study and the inconsistency of chemotherapy regimen.

The toxicity of apatinib in monotherapy and combined therapy in this study were mild and manageable. Hand-foot syndrome, hypertension, proteinuria, mucositis, anorexia, fatigue were the most frequently observed adverse events in current study. Incidence of hand-foot syndrome and hypertension was similar to Miao’s study [21]. Consistent with most common adverse events in Lan’s study [20], proteinuria, mucositis, anorexia and fatigue were frequently happened and relatively severe. This study treatment regime included apatinib monotherapy and apatinib combined with chemotherapy, and discrepancy in patients basic condition and tolerance for drug contributed to the different adverse reactions between studies.

The National Comprehensive Cancer Network (NCCN) guidelines for OC suggest that biochemical recurrent patients may: 1) enroll in a clinical trial; 2) delay treatment until clinical relapse; 3) receive immediate platinum-based recurrence therapy; or 4) undergo best supportive care [29]. Thus, several studies aimed to investigate low-toxicity agents to delay the appearance of measurable disease in these patients [30]. However, no efficacy agent was confirmed until now. In our study, we demonstrated the efficacy of apatinib in biochemical recurrent OC patients with a median PFS of 6 months. This result implied that early treatment using apatinib in biochemical-only recurrent OC may extend time to clinical disease progression and delay time to intravenous chemotherapy, with low toxicity. However, a large sample study is needed to confirm the effect of apatinib in biochemical relapse.

One of the potential shortcomings of this study is that it was a relatively small-scale retrospective study; only 19 biochemical recurrent patients were evaluable. Prospective studies on a large sample cohort are needed to confirm the value of apatinib for the treatment of biochemical relapse patients. Additionally, this study failed to find a biomarker to predict the efficacy in biochemical recurrent patients. In this study, patients experienced similar grades of AEs to previous studies of apatinib treatment in OC. Hand-foot syndrome, mucositis, fatigue, anorexia, proteinuria, and hypertension were the most common adverse effects; however, all were tolerable.

## Conclusions

In this study, we focused on the activity of apatinib in biochemical recurrent OC patients. Results indicate that apatinib might be promising for such patients to delay clinical disease progression and intravenous chemotherapy. More important, our study brings the potential breakthrough for treating OC patients with asymptomatic biochemical relapse.

## Data Availability

The datasets used and analyzed in the current study are available from the corresponding author upon reasonable request.

## Abbreviations

OC: Ovarian Cancer
CR: Complete Remission
PR: Partial Remission
SD: Stable Disease
PD: Progressive Disease
ORR: Objective Response Rate
DCR: Disease Control Rate
PFS: Progression-Free Survival
AE: Adverse Event
